# Composition of Platelet-Rich Plasma Prepared From Knee Osteoarthritic Patients: Platelets, Leukocytes, and Subtypes of Leukocyte

**DOI:** 10.7759/cureus.36399

**Published:** 2023-03-20

**Authors:** Thana Turajane, Vorasilp Cheeva-akrapan, Pamok Saengsirinavin, Wanpen Lappaiwong

**Affiliations:** 1 Biotechnological Research and Development Center, Police General Hospital, Bangkok, THA; 2 Orthopedic Surgery, Police General Hospital, Bangkok, THA

**Keywords:** neutrophil-leukocyte, leukocyte-rich prp, plasma therapy, knee osteoarthritis/ koa, platelet-rich plasma/ prp

## Abstract

Introduction: Platelet-rich plasma (PRP) has gained increasing popularity in the orthopedic field. There has been still no consensus on PRP preparation technique, thus providing a variety of final PRP products. Different preparation techniques lead to different compositions of PRP, which include platelet concentration, the number of leukocytes, and their subtypes. Here, we studied those compositions of PRP compared to whole blood samples.

Methods: There were 335 participants who met the inclusion and exclusion criteria. Each participant underwent a blood drawing process to prepare PRP for their knee osteoarthritis treatment. We categorized baseline platelet concentration in whole blood samples into three groups: less than 2 x 10^5^/µL (Group 1), between 2 x 10^5^/µL and 2.99 x 10^5^/µL (Group 2), and greater than 2.99 x 10^5^/µL (Group 3). The primary outcome was reported as the platelet concentration in PRP and the ratio of platelet concentration in PRP compared to baseline whole blood samples. The secondary outcome was reported as the ratios of leukocyte, lymphocyte, and neutrophil in PRP compared to the baseline whole blood samples.

Results: The average platelet concentration in PRP was 1.26 x 10^6^/µL (6.3 times higher compared to baseline whole blood samples). The mean platelet concentration of PRP in Group 1, Group 2, and Group 3 was 1.08 x 10^6^/µL, 1.38 x 10^6^/µL, and 1.71 x 10^6^/µL, respectively (p-value = 0.0001). The platelet concentration ratio of PRP condition to the baseline whole blood was 6.9, 5.8, and 4.2 in Group 1, Group 2, and Group 3, respectively (p-value = 0.0018). The average ratio of leukocytes in PRP to whole blood was 1.5. The average ratio of lymphocytes and neutrophils in PRP to whole blood was 2.0 and 0.5, respectively.

Conclusion: Different baseline platelet concentrations in whole blood samples provided significantly different platelet concentrations in PRP. The baseline platelet concentration in whole blood also provided an inverse relation to the fold change of the platelet concentration in PRP. Subtypes of leukocytes changed from neutrophil-predominated in the baseline whole blood samples to lymphocyte-predominated in PRP.

## Introduction

Platelet-rich plasma (PRP) is an autologous blood derivative prepared from a centrifugation process that concentrates platelet, removes red blood cells, and provides different leukocytes composition depending on preparation techniques [1]. With the supra-physiologic concentration of platelets, PRP provides numerous growth factors and cytokines from α-granules of platelets. These growth factors and cytokines include platelet-derived growth factor (PDGF), transforming growth factor-β (TGF-β), vascular endothelial growth factor (VEGF), and insulin-like growth factor-1 (IGF-1) [2-6]. These molecules exert their action both individually and synergistically to enhance cellular migration, proliferation, and angiogenesis which are important steps in tissue inflammation and regeneration [7,8]. PRP application has diversified among orthopedics to treat the pathology of osteocytes, chondrocytes, and tendinocytes [9,10].

Different preparation techniques lead to different components of PRP. To our knowledge, there is still no report on the optimal platelet concentration for PRP [11]. Recent research has focused on leukocyte composition in PRP regarding the controversial inflammatory properties. The buffy coat preparation technique provides leukocyte-rich PRP (LR-PRP), whereas the plasma-based technique provides leukocyte-poor PRP (LP-PRP) [12]. A disadvantage of a high concentration of leukocytes in PRP is the increase in the expression of catabolic cascades and inflammatory markers such as interleukin-1 (IL-1) and tumor necrosis factor-α (TNF-α). However, LR-PRP is thought to have the IL-1 receptor antagonist protein (IL-1RAP), which blocks the IL-1 and supports the healing cascade [13]. Some studies reported the advantages of leukocytes in PRP in terms of antibacterial and immunological resistance [14]. Due to the incongruent results of LR-PRP and LP-PRP usage, some authors proposed that different compositions of leukocytes could also affect the efficacy of PRP [7].

The primary outcome of this current study is to evaluate the components of PRP in terms of platelet concentration and the ratio of platelet concentration in PRP compared to the baseline whole blood samples. We hypothesized that different baseline platelet concentration provides different fold change in platelet concentration in PRP. The secondary outcome is to evaluate the ratios of leukocyte, lymphocyte, and neutrophil in PRP compared to the baseline whole blood samples.

## Materials and methods

A cross-sectional analytic study was conducted, after the Institutional Review Board and the Police General Hospital’s Ethics Committee approval (approval number: 49/2019), from June 2020 to October 2021 at the Biotechnological Research and Development Centre, Police General Hospital, Bangkok, Thailand. Knee osteoarthritic patients who were eligible for PRP treatment at our institution’s orthopedic clinic were identified. Inclusion criteria were the following: diagnosis of knee osteoarthritis classified as Kellgren and Lawrence (KL) I-IV and platelet concentration in whole blood > 80,000 cells/µL. Exclusion criteria were the following: history of using non-steroidal anti-inflammatory drugs (NSAIDs) within five days of blood drawn or taking anticoagulant or anti-aggregate drugs. The rationale behind these criteria was to study the hematologic profile of the representative groups that are normally treated with PRP treatment. The hematologic profile may be different from those of healthy participants typical in previous studies.

A total of 335 participants met the criteria. All participants were informed about the study protocol and provided with informed consent.

PRP preparation protocol 

**Figure 1:**
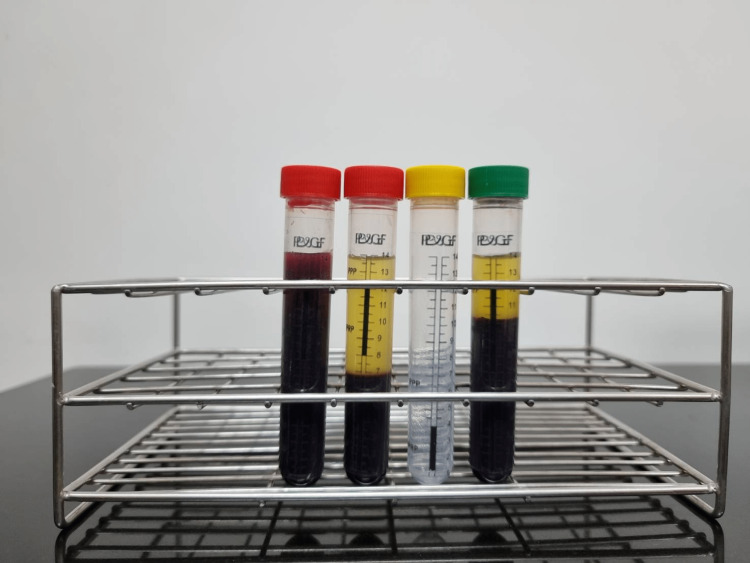
PRP preparation tubes. The red tubes are for the first spinning process. The yellow tube is for the second spinning process. The green tube is for platelet-rich fibrin preparation. PRP: platelet-rich plasma

A 30-ml peripheral blood sample for a single knee injection (60 ml for bilateral knees) was collected from each participant. A complete blood count was performed at the initial blood draw. The first 20 ml of blood was divided into two centrifuge tubes (red) (PP&GF, Bangkok, Thailand) (Figure 1) containing 10 ml each to mix with acid citrate dextrose anticoagulant. The blood was centrifuged using the ALPAS centrifugation machine (ALPAS, Bangkok, Thailand) at 250 g for six minutes. After the first spin, the blood was separated into three components: red blood cells at the bottom layer, a buffy coat in the middle layer, and platelet-containing plasma at the top layer. The upper two layers were gently aspirated and transferred to a new tube (yellow) and centrifuged again at 1000 g for 10 minutes. After the second spin, the platelet-poor supernatant plasma was gently aspirated for removal. One milliliter of residual LR-PRP was collected for a complete blood count. The remaining PRP was aspirated using a 5-ml sterile injection syringe for intra-articular injection. The remaining 10 ml of peripheral blood was transferred to the last tube (green) and centrifuged once with 250 g for six minutes to produce platelet-rich fibrin (PRF) and to be used as a natural activator. All procedures were performed in an A-class sterile biosafety cabinet.

Hematological analysis

Platelet concentration (total number of platelets/mL), total leukocyte count, lymphocyte percentages, and neutrophil percentages from whole blood and PRP were determined using an automated hematology analyzer (URIT-3000Plus, URIT Medical Electronic, Guangxi, China) immediately after preparation.

Statistical analysis 

The sample size calculation was calculated for a one-way analysis of variance (ANOVA) test. The effect size was 0.4. The α error and β error were 0.05 and 80%, respectively. The total sample size was 159 (53 in each group). Continuous data were elaborated into mean and standard deviation. Discrete data were elaborated into percentages and proportions. We displaced the data to evaluate the distribution by histogram. If the data were normally distributed, the ANOVA test was used to test the difference in the variance. If the data were not normally distributed, Kruskal-Wallis was used to determine the statistically significant difference. The two-tail test was used. We considered a p-value of < 0.008333 significant for the ratio outcome, and a p-value of < 0.05 significant for non-ratio data. We used Stata software, version 16.0 (2019; StataCorp LLC, College Station, Texas, United States), for statistical analysis.

## Results

A total of 335 participants was included in the study. Among these, 129 participants (38.51%) were female, and 206 participants (61.49%) were male. The ages of participants ranged from 50 years to 93 years with a mean age of 65 years. The average platelet concentration in whole blood samples was 2.08 x 10^5^/µL (range, 0.89 x 10^5^ to 4.53 x 10^5^). We categorized baseline platelet concentration in whole blood samples into three groups according to the classification proposed by Kon et al. [15]: less than 2 x 10^5^/µL (Group 1), between 2 x 10^5^/µL and 2.99 x 10^5^/µL (Group 2), and greater than 2.99 x 10^5^/µL (Group 3). There were 141, 188, and six specimens in Group 1, Group 2, and Group 3, respectively. Baseline characteristics (subject age, baseline leukocyte count, baseline percentage of lymphocyte, and baseline percentage of neutrophil) in each group were not statistically significantly different (p-value = 0.56, 0.19, 0.58, and 0.13, respectively). However, sex in each group was statistically significantly different (p-value < 0.001) with the male predominant in Group 1 and female predominant in Group 2 and Group 3 (Table 1).

**Table 1:** Baseline characteristic data of all samples and in each group according to baseline platelet concentration in whole blood samples. A p-value < 0.05 was considered significant.

	Overall	Group 1	Group 2	Group 3	The p-value for subgroup analysis
Baseline platelet concentration (/µL; Mean ± S.E.)	2.08 x 10^5^ ± 0.02 x 10^5^	1.59 x 10^5^ ± 0.02 x 10^5^	2.38 x 10^5^ ± 0.01 x 10^5^	3.93 x 10^5^ ± 0.28 x 10^5^	-
Age (year; Mean ± S.E.)	64.8 ± 0.4	65.1 ± 0.7	64.5 ± 0.6	64 ± 2.4	0.56
Number of specimens	335	141	188	6	-
Sex (#Female/#Male)	129/206	27/114	98/90	4/2	< 0.001
Baseline leukocyte count (/µL; Mean ± S.E.)	6,132.3 ± 73.6	6,128.4 ± 104.7	6,103.2 ± 103.7	7,133.3 ± 450.7	0.18
Baseline percentage of lymphocyte (%; Mean ± S.E.)	29.4 ± 0.5	29.8 ± 0.7	29.2 ± 0.6	26.6 ± 2.7	0.58
Baseline percentage of neutrophil (%; Mean ± S.E.)	59.6 ± 0.6	60.9 ± 0.9	58.7 ± 0.8	60 ± 4.6	0.13

**Figure 2:**
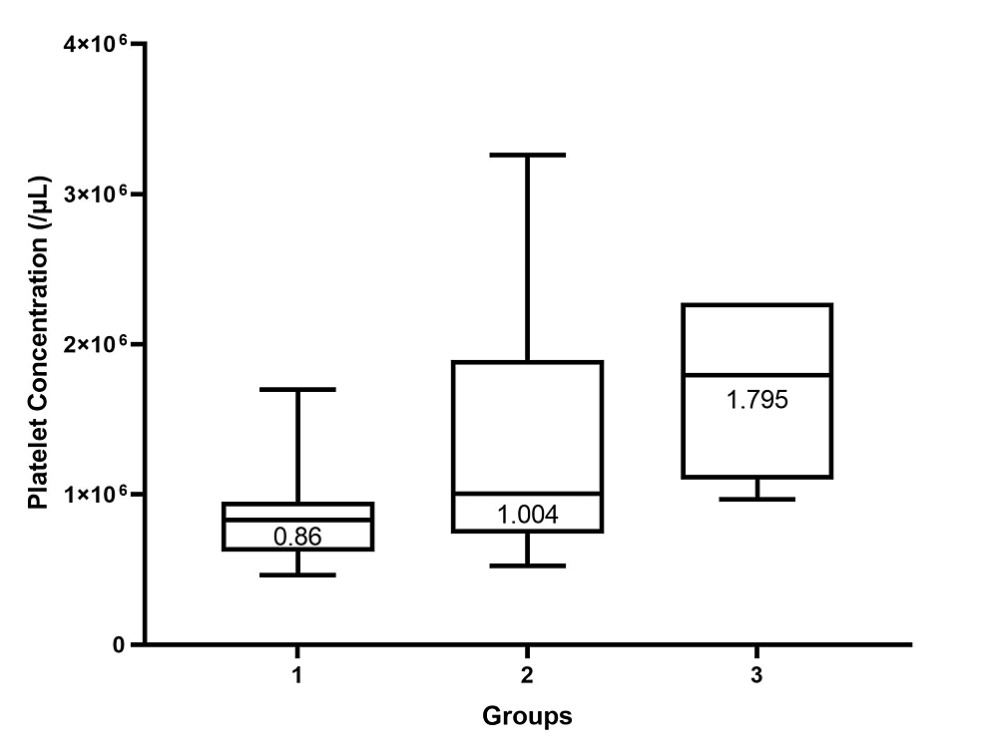
Box plot showing platelet concentration in PRP categorized by groups according to the baseline platelet concentration in whole blood. The median platelet concentration in the PRP of each group is shown. The Kruskal-Wallis test was used to test the significant difference in platelet concentration in PRP among the three groups. Platelet concentration in the PRP among the three groups was statistically significantly different (p-value = 0.0001). Comparisons between every two groups were also done. A statistically significant difference was found between groups 1 and 2, and groups 1 and 3 (p-value < 0.0001, = 0.005, respectively). Comparison between groups 2 and 3 did not show a statistically significant difference (p-value = 0.07). We considered a p-value of < 0.05 significant. PRP: platelet-rich plasma

The average platelet concentration in PRP was 12.6 x 10^5^/µL (range, 4.6 x 10^5^ to 32.6 x 10^5^), 10.8 x 10^5^/µL (range, 4.64 x 10^5^ to 29.9 x 10^5^), 13.8 x 10^5^/µL (range, 5.23 x 10^5^ to 32.6 x 10^5^), and 17.1 x 10^5^ (range, 9.67 x 10^5^ to 22.8 x 10^5^) for overall samples, Group 1, Group 2, and Group 3, respectively. Platelet concentrations among the subgroups were significantly different (p-value < 0.0001) (Table 2). When comparing the two groups, platelet concentration in PRP between groups 1 and 2, and groups 1 and 3 were statistically significantly different (p-value < 0.0001, = 0.005, respectively). However, platelet concentration in PRP between groups 2 and 3 was not statistically significantly different (p-value = 0.07), as seen in Figure 2.

**Table 2:** Primary and secondary outcome of overall samples and each group according to baseline whole blood platelet concentration. We considered a p-value of < 0.008333 significant for ratio data and a p-value of < 0.05 significant for non-ratio data. PRP: platelet-rich plasma

	Overall	Group 1	Group 2	Group 3	The p-value for the difference of subgroup analysis
Platelet concentration in PRP (/µL; Mean ± S.E.)	1.26x10^6^ ± 0.04x10^6^	1.08x10^6^ ± 0.05x10^6^	1.38x10^6^ ± 0.06x10^6^	1.71x10^6^ ± 0.26x10^6^	0.0001
Ratio of platelet concentration in PRP to baseline whole blood sample (fold change; Mean ± S.E.)	6.3 ± 0.2	6.9 ± 0.4	5.8 ± 0.3	4.2 ± 0.4	0.0018
Ratio of leukocyte in PRP to baseline whole blood sample (fold change; Mean ± S.E.)	1.5 ± 0.03	1.5 ± .0.1	1.5 ± 0.1	1.2 ± 0.1	0.21
Percentage of lymphocyte (%, Mean ± S.E.)	54.7 ± 0.7	54.7 ± 1.1	54.7 ± 0.9	53.6 ± 6.2	0.84
Ratio of lymphocyte in PRP to whole blood sample (fold change; Mean ± S.E.)	2.0 ± 0.03	1.9 ± .0.1	2.0 ± 0.04	2.1 ± 0.3	0.76
Percentage of neutrophil (%, Mean ± S.E.)	29.0 ± 0.9	29.4 ± 1.5	28.8 ± .1.1	22.6 ± 5.6	0.47
Ratio of neutrophil in PRP to whole blood sample (fold change, Mean ± S.E.)	0.5 ± 0.02	0.5 ± .0.03	0.5 ± 0.02	0.4 ± 0.1	0.14

**Figure 3:**
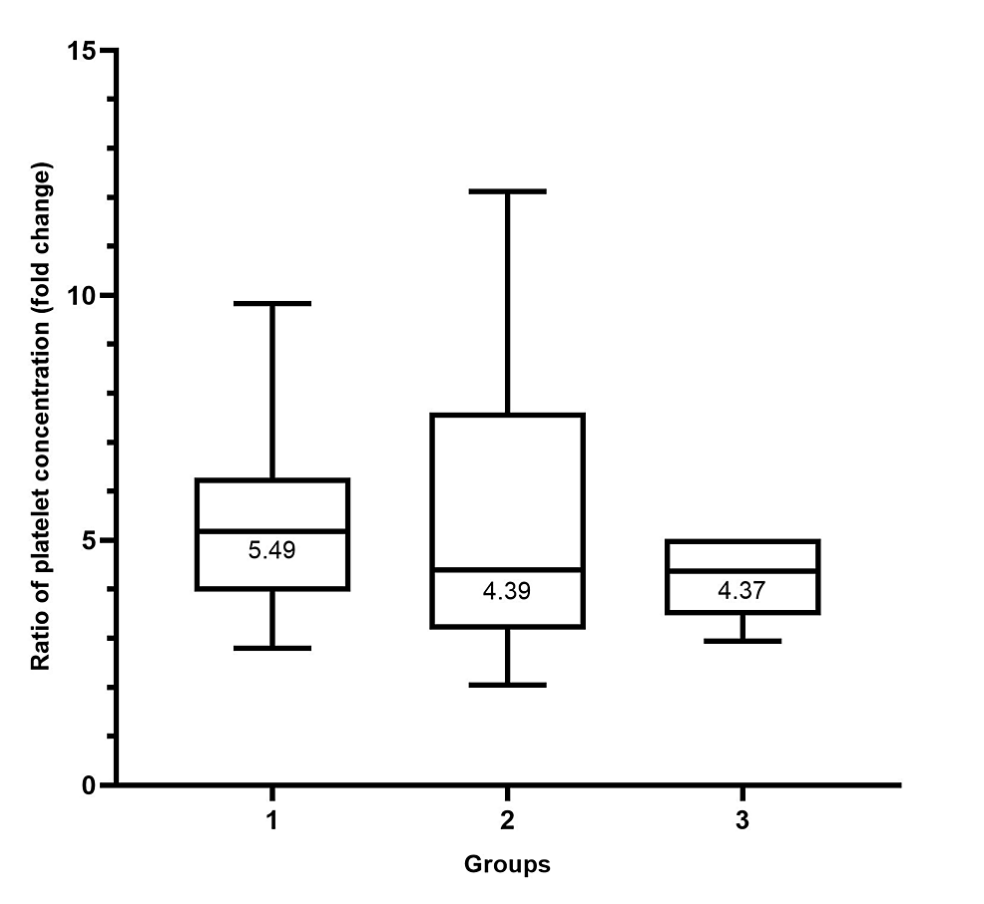
Box plot of the ratio of platelet concentration in PRP to baseline whole blood samples categorized by groups according to baseline whole blood platelet concentration. The median of the ratio of platelet concentration in each group is shown. The Kruskal-Wallis test was used to test the significance among the three groups. The ratio of platelet concentration among three groups was statistically significantly different (p-value = 0.0018). Comparisons between every two groups were also done. A statistically significant difference was found between groups 1 and 2 (p-value = 0.0004). Comparisons between groups 1 and 3 and groups 2 and 3 were not found to be statistically significantly different (p-value = 0.05, = 0.228 respectively). We considered a p-value of < 0.008333 significant. PRP: platelet-rich plasma

The average ratio of platelet concentration in PRP to baseline whole blood samples was 6.3 (range, 2.1 to 21.5), 6.9 (range, 2.8 to 21.5), 5.8 (range, 2.1 to 14.9), and 4.2 (range, 2.9 to 5.0) for overall samples, groups 1, 2, and 3, respectively. There was a statistically significant difference in the ratio of platelet concentration among the three groups (p-value = 0.0018) (Table 2). When comparing two groups, the ratio of platelet concentration between groups 1 and 2 was statistically significantly different (p-value = 0.0004). However, the ratio of platelet concentration between groups 1 and 3, and groups 2 and 3 were not statistically significantly different (p-value = 0.05, 0.228, respectively), as seen in Figure 3.

**Figure 4:**
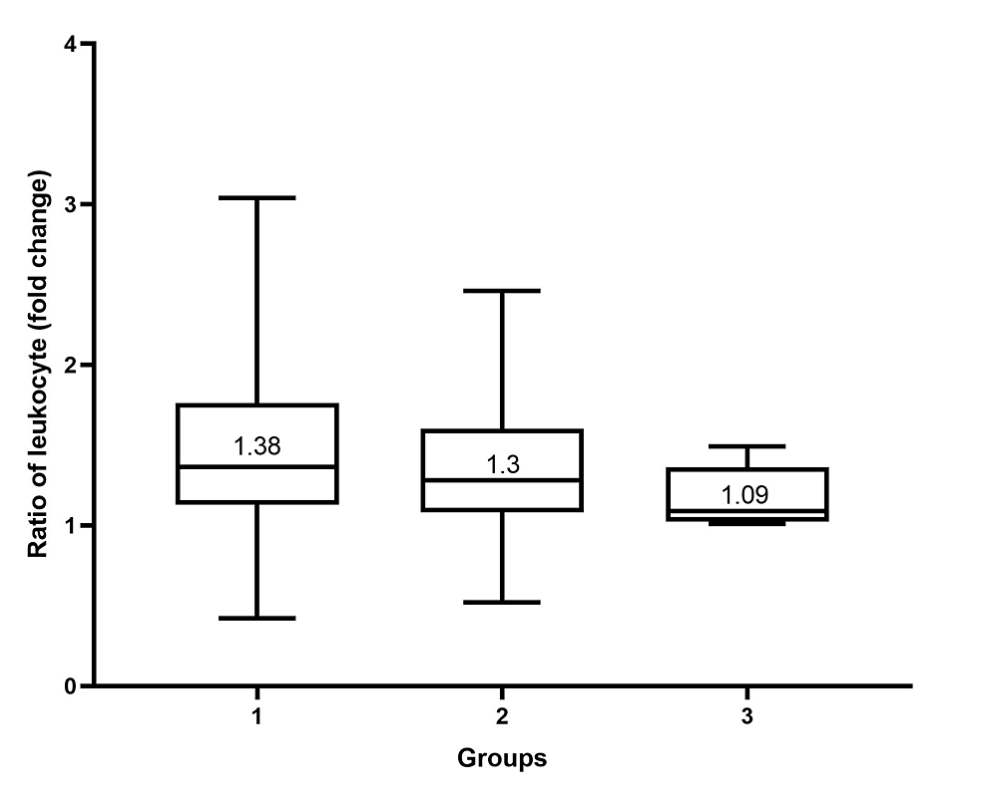
Box plot showing the ratio of leukocytes in PRP to baseline whole blood samples categorized by groups according to baseline whole blood platelet concentration. The median of the ratio of leukocytes in each group is shown. The Kruskal-Wallis test was used to test the significance among the three groups. The ratio of leukocytes among the three groups was not statistically significantly different (p-value = 0.21). We considered a p-value of < 0.008333 significant. PRP: platelet-rich plasma

**Figure 5:**
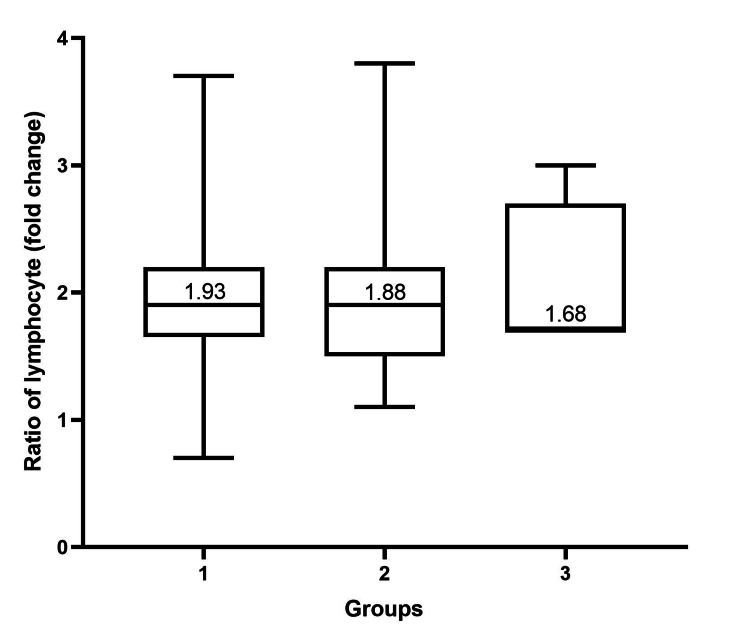
Box plot showing the ratio of lymphocytes in PRP to baseline whole blood samples categorized by groups according to baseline whole blood platelet concentration. The median of the ratio of lymphocytes in each group is shown. An ANOVA test was used to determine the significance among the three groups. The ratio of lymphocytes among the three groups was not statistically significantly different (p-value = 0.76). We considered a p-value of < 0.008333 significant. PRP: platelet-rich plasma

**Figure 6:**
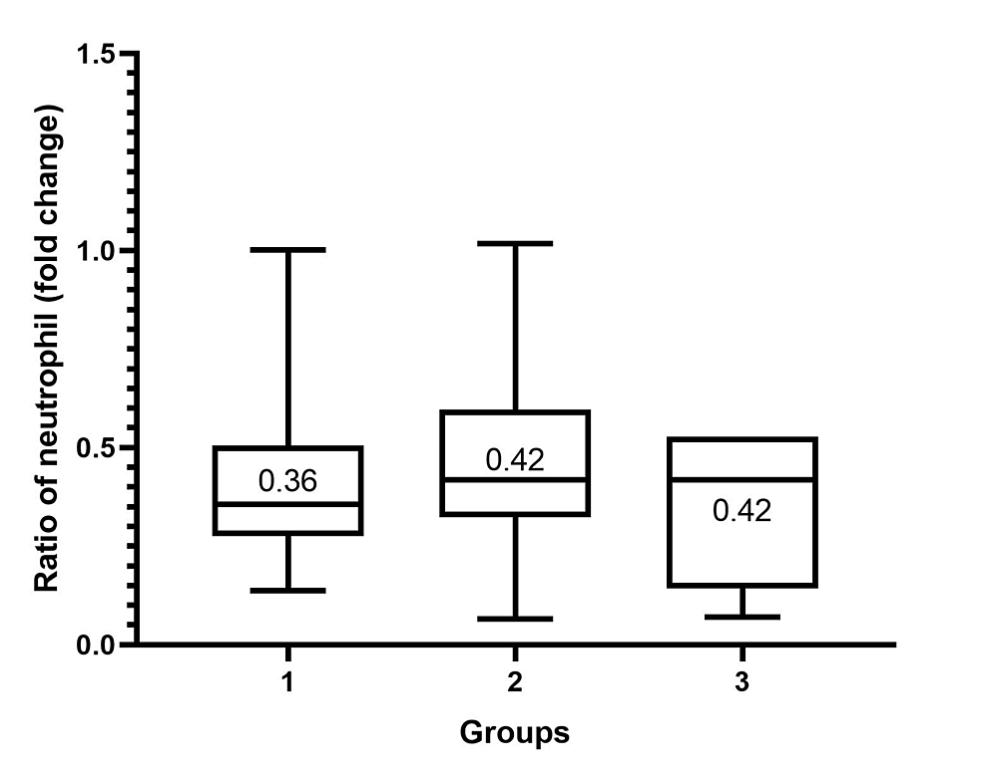
Box plot showing the ratio of neutrophils in PRP to baseline whole blood samples categorized by groups according to baseline whole blood platelet concentration. The median of the ratio of neutrophils in each group is shown. The Kruskal-Wallis test was used to test the significance among the three groups. The ratio of neutrophils among the three groups was not statistically significantly different (p-value = 0.14). We considered a p-value of < 0.008333 significant. PRP: platelet-rich plasma

To study the changes in the hematological profile of PRP, we compared those profiles in PRP with the baseline whole blood. The average ratio of leukocytes in PRP to whole blood was 1.5 (range, 0.4 to 5.4). The average percentage of lymphocytes in PRP was 54.69% (range, 21.9 to 78.4). The average ratio of lymphocytes in PRP to whole blood was 2.0 (range, 0.7 to 4.0). The average percentage of neutrophils in PRP was 28.9% (range, 2.7 to 71.9). The average ratio of neutrophils in PRP to whole blood was 0.5 (range, 0.7 to 1.3). We also performed a subgroup analysis of each parameter according to groups. There was no statistically significant difference in each parameter (Figures 4, 5, 6) (Table 2).

## Discussion

PRP application in orthopedics has gained increasing popularity. Many researchers are studying the PRP composition for their highest efficacy. To our knowledge, this is one of the first studies on PRP composition that has a large sample size (335 specimens) from knee osteoarthritic patients.

We found that different baseline platelet concentration in whole blood samples provides a statistically significant difference in platelet concentration in PRP. We also observed that baseline platelet concentration in whole blood was inversely correlated to the fold change of platelet concentration in PRP products. We proposed that the platelet concentration of PRP products would reach a plateau level to prevent excessively concentrated PRP. This is supported by Weibrich et al. in 2004, who stated that highly concentrated platelets had an inhibitory effect on osteoblast activity when compared to lower concentrations [16]. Further study of the relationship between platelet concentration in PRP and the clinical outcome of treatment should be conducted.

Leukocytes originate from myeloid and lymphoid cells. Myeloid stem cells give rise to neutrophils, eosinophils, basophils, and monocytes. Lymphoid stem cells give rise to lymphocytes, which further divide into B cells, T cells, and natural killer cells [12]. It remains controversial whether LR-PRP or LP-PRP provide the better clinical outcomes regarding the leukocyte content in PRP. Many in vitro studies showed that LR-PRP has a large effect on inflammation. However, the finding is different in vivo. Mariani et al. in 2016 demonstrated that LR-PRP did not upregulate pro-inflammatory cytokines in vivo [17]. These controversial findings highlight the complexity of leukocyte role in PRP treatment.

We used LR-PRP. Buffy coat-based PRP preparation in this study protocol provided LR-PRP in which the leukocyte content in PRP increased 1.5 fold from the baseline whole blood. One of the proposed ideas that favor the use of LR-PRP for orthopedics treatment is “macrophage polarization,” according to which peripheral blood mononuclear cells including lymphocytes, natural killer cells, and monocytes transform into macrophages when merging into the tissue. Tissue macrophages then interact reciprocally with activated T&B lymphocytes. Macrophages phenotypically express themselves in two forms: M1 macrophages, which act mainly as pro-inflammatory cells, and M2 macrophages, which act mainly as anti-inflammatory cells. Macrophages can polarize between these two subtypes according to the microenvironment. M1 macrophages usually function in the early phase of healing while M2 macrophages usually function in the late phase of healing. With these functions, macrophages provide a protective immunological role by promoting angiogenesis through growth factors and cytokines. This theory supports that different components of leukocytes in PRP may play an important role in their clinical efficiency [18]. Lymphocytes play an indirect role in the tissue healing process by steering monocyte differentiation from M1 pro-inflammatory subtypes to M2 anti-inflammatory subtypes. T lymphocyte-derived cytokines including interferon-γ (IFN-γ) and interleukin-4 (IL-4) were proven responsible for this process [19]. Weirather et al. in 2014 performed an in vivo study to demonstrate that T lymphocytes indirectly aid in tissue healing in a mouse model by modulating monocyte and macrophage differentiation [20]. These mechanisms highlight the strength of higher lymphocytes in LR-PRP in this current study.

Neutrophils generally serve as pro-inflammatory mediators and are thought to promote catabolic effects. They release inflammatory cytokines and matrix metalloproteinases, which lead to collagen degradation and fibrosis [7,21-23]. The reduction in neutrophil population in PRP compared to whole blood was found in many studies [22,24]. Kobayashi et al. in 2016 found that the inhibition of platelet-neutrophil interaction could lead to an increase in TGF-β, which is responsible for collagen synthesis [25]. Harrison et al. in 2021 proposed that the decrease in neutrophil population may be due to the high density of neutrophils in pre-centrifuged blood that traps neutrophils in the red blood cell layer when PRP is being processed by centrifugation. [26]

One of the interesting findings in this study is the change in the majority of the composition of leukocytes in PRP products compared to those in whole blood. In baseline whole blood, neutrophils predominate at 59.6%, whereas lymphocytes constitute only 29.4%. In PRP, the neutrophil population decreased to 29% and lymphocytes predominated at 54.7%. The lymphocyte percentage in PRP increased two-fold from baseline whole blood, whereas the neutrophil percentage in PRP decreased 0.5-fold from the baseline whole blood. These findings correlated with many previous studies that found lymphocytes predominated over neutrophils in platelet-rich products [8,22,24]. The finding that LR-PRP predominated with lymphocytes instead of neutrophils may be one of the key answers for the usage of LR-PRP over LP-PRP. This also highlights the importance of classification for PRP to include a subtype of leukocytes for the best clinical application.

There are limitations to this study. First, there was no correlation study between the blood component and the clinical outcome of the patient. With further study, researchers may be able to determine the optimal level of platelet, leukocyte, and subtypes of leukocyte concentration in PRP for the treatment efficacy in specific tissues. Future researchers should study the relationship between subtypes of leukocytes and the level of growth factors and cytokine in PRP with clinical effectiveness. Second, PRP in this current study was prepared from one PRP preparation technique. Different PRP preparations may provide different outcomes on their composition. Third, the automated hematology analyzer used in this study did not provide a monocyte profile, which limited the variety of subtypes of leukocytes. Fourth, we were unable to reach the calculated sample size in Group 3 due to the rarity of platelet concentration higher than 2.99 x 10^5^/µL in the normal population.

## Conclusions

Our study found that different baseline platelet concentrations in whole blood provided significantly different platelet concentrations in PRP. The baseline platelet concentration in whole blood also provided an inverse relation to the fold change of platelet concentration in PRP. We also found that lymphocytes predominated over neutrophils in PRP.
